# Mental health status in veterans residing in rural versus non-rural areas: results from the veterans’ health study

**DOI:** 10.1186/s40779-020-00272-6

**Published:** 2020-09-21

**Authors:** Joseph J. Boscarino, Charles R. Figley, Richard E. Adams, Thomas G. Urosevich, H. Lester Kirchner, Joseph A. Boscarino

**Affiliations:** 1 Clinical Psychology Department, William James College, Newton, MA 02459 USA; 2 School of Social Work, Tulane University, New Orleans, LA 70112 USA; 3 Department of Sociology, Kent State University, Kent, OH 44242 USA; 4 Ophthalmology Service, Geisinger Clinic, Mount Pocono, PA 18344 USA; 5 Department of Population Health Sciences, Geisinger Clinic, 100 N. Academy Avenue, 44-00, Danville, PA 17822 USA

**Keywords:** Veterans, Rural, Risk factors, PTSD, Alcohol abuse, Depression, Service use, Census data

## Abstract

**Background:**

The majority of Veterans Affair (VA) hospitals are in urban areas. We examined whether veterans residing in rural areas have lower mental health service use and poorer mental health status.

**Methods:**

Veterans with at least 1 warzone deployment in central and northeastern Pennsylvania were randomly selected for an interview. Mental health status, including PTSD, major depression, alcohol abuse and mental health global severity, were assessed using structured interviews. Psychiatric service use was based on self-reported utilization in the past 12 months. Results were compared between veterans residing in rural and non-rural areas. Data were also analyzed using multivariate logistic regression to minimize the influence by confounding factors.

**Results:**

A total of 1730 subjects (55% of the eligible veterans) responded to the survey and 1692 of them had complete geocode information. Those that did not have this information (*n =* 38), were excluded from some analyses. Veterans residing in rural areas were older, more often of the white race, married, and experienced fewer stressful events. In comparison to those residing in non-rural areas, veterans residing in rural areas had lower global mental health severity scores; they also had fewer mental health visits. In multivariate logistic regression, rural residence was associated with lower service use, but not with PTSD, major depression, alcohol abuse, and global mental health severity score after adjusting confounding factors (e.g., age, gender, marital status and education).

**Conclusions:**

Rural residence is associated with lower mental health service use, but not with poor mental health in veterans with former warzone deployment, suggesting rural residence is possibly protective.

## Background

Significant numbers of US military personnel experience post-deployment depression, posttraumatic stress disorder (PTSD), and substance use disorders upon return from warzone deployment [1–5]. A previous study suggested that the burden of providing care for veterans with mental disorders is greater in rural settings [6]. Increasingly, military recruits in the US have been drawn from rural areas [7], raising the potential mental health burden in these geographic regions [8]. In contrast, VA facilities tend to be located within larger population centers [6].

Compared to urban and suburban veterans, rural veterans tend to visit their health providers less frequently, have access to fewer mental health and specialty services, and may have more physical and mental health problems [6, 9, 10]. Given these factors, poorer quality mental health care is likely more pronounced in the rural setting [11]. Also, many veterans do not use VA health care services [2, 12]. Furhermore, non-VA providers in rural areas may lack the training and experience to manage PTSD and related disorders among veterans [6, 13]. Past research has suggested that the provision of mental health services for veterans in rural areas has improved over time, and some health disparities previously reported between rural and non-rural veterarns were often neither clinically significant nor universal to all veterans across VA and non-VA settings [9, 14]. In addition, the implementation of the “VA Choice” program may have expanded service options for veterans, which in turn, may have also improved service provision in rural areas [14]. The Choice program allows veterans to receive VA-related health care from civilian providers in the community. This program was designed to address VA’s delays in providing medical care. Currently, VA Choice is being replaced by the “Mission Act” program, which may have an even greater impact in the care for veterans in the future [15]. The Mission Act program focuses on provision of better family and caregiver support for veterans injured in the line of duty on or after September 11, 2001, but may expand to other veteran groups in the future. Thus, the post-deployment health status of veterans residing in rural areas of the US is currently unclear. Given the nature of current international conflicts, concerns related to provision of mental health services for veterans in rural areas is relevant in the US as well as many other parts of the world [16].

Most US veterans today have private health insurance and/or Medicare coverage and receive at least some or most of their care from non-VA institutions [17–20]. Thus, it is important to study veterans in the community and not just those seen in government facilities [2]. The availability of different health care options for veterans will likely increase, so non-VA health services may require evidence-based modification for better facility planning. Because of the expanding service options available for US veterans [2, 16], definitive data sources related to service use and health status for veterans are limited [21, 22]. The knowledge gained from studying veterans in non-VA health care settings is important for monitoring the quality of care received by all veterans in a more generalizable manner, especially among those who are generally less likely to use the VA health care system [22, 23]. Most US veteran populations are community based and study samples drawn from VA hospitals and military clinics are biased in that they likely overrepresent treatment-seeking veterans in more urban areas [24]. Consequently, the current study is conducted to assess the impact of recent service changes that may have differentially affected rural areas [25]. At this time, the consensus is that, at least for the near future, multiple data sources (both VA and non-VA) will be required to study post-deployment health outcomes among US veterans [2, 24].

## Methods

### Study population

**Fig. 1:**
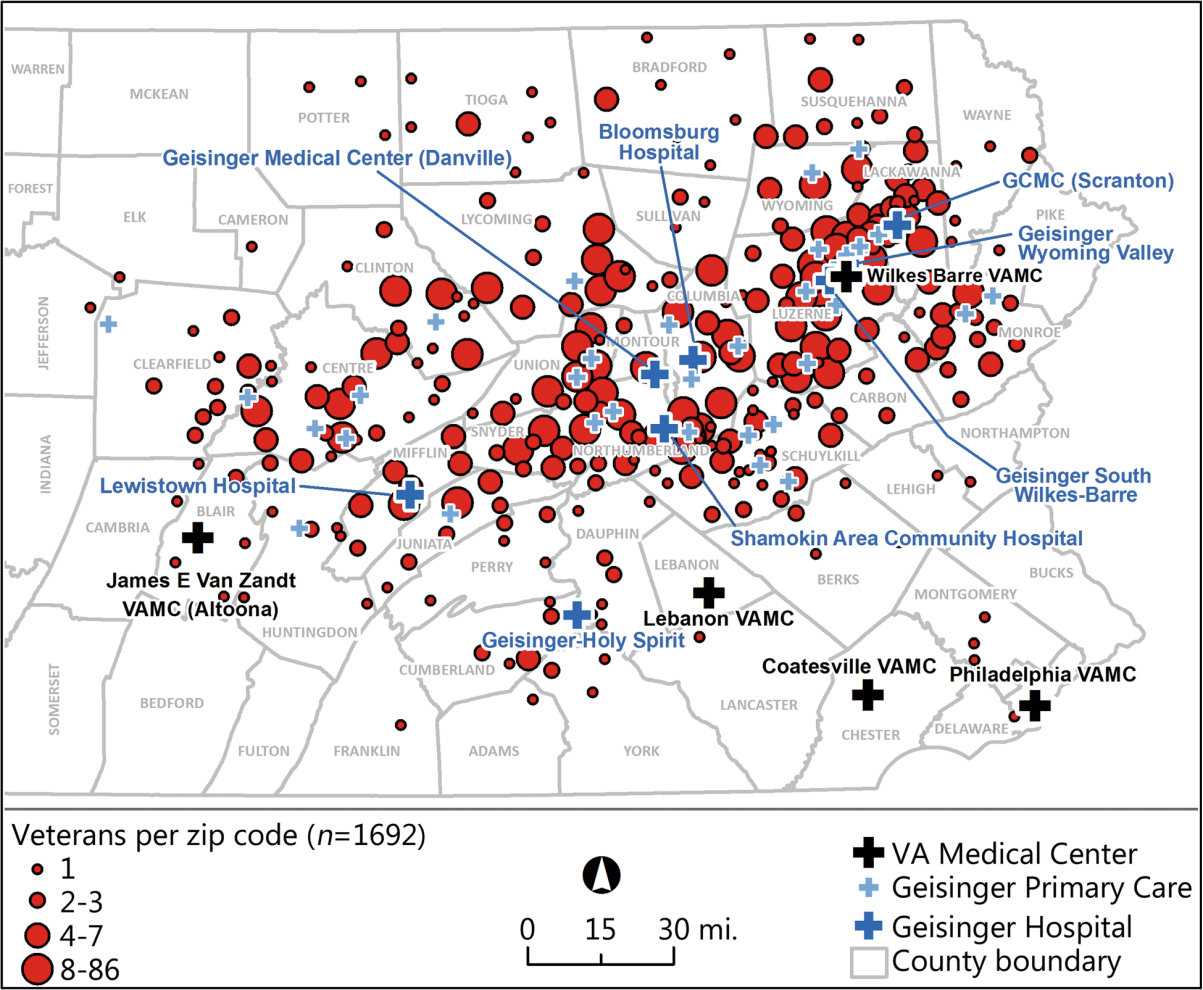
Veterans surveyed in service area, Local VA Hospitals, and Geisinger’s Hospitals/Clinics (38 veterans had missing zipcode data and excluded from geographic analyses, *N =* 1692)

This study is based on a random cross-sectional telephone survey of community-based US military veterans recruited for a study of the health effects of military service [1, 26, 27]. All veterans in the study were outpatients in the Geisinger Clinic, the largest multi-hospital system in central and northeastern Pennsylvania [1]. Geisinger is an integrated health services organization with an advanced electronic health record system (www.geisinger.org). This system serves more than 3 million residents throughout 45 counties in central, south-central and northeast Pennsylvania and encompasses a 25,000 mile^2^ (40,000 km^2^) heavily rural service area (Fig. 1). Geisinger is a large regional managed care system that accepts all payers, including manage care clients, Medicare, Medicaid, private insurance, Tricare, VA, Worker Compensation, among others [28]. The overall insurance mix for patients in the current study was 25% commercial insurance, 28% managed care plans, 30% Medicare, 12% VA/Tricare coverage, and 4% other payers. The surveys for this study were conducted from Febuary, 2016 through Febrary, 2017 [1]. This study was approved by both the Institutional Review Boards of the Geisinger Clinic (IRB Study #2015–0441) and the US Department of Defense (IRB Study #A-18989). All patients provided their informed consent for this study and were offered a small monetary incentive for participation.

### US census data

Patients were geocoded using US Census zip-code tabulation area (ZCTA) data (www.census.gov/geo/reference/zctas.html). ZCTAs are generalized area representations of the United States Postal Service (USPS) zip code areas. The frequent zip code change by the USPS is usually not reflected in the annual Census Bureau updates. Each ZCTA is constructed by aggregating US Census blocks whose addresses use a given ZIP code. In assembling census statistical units to create ZCTAs, the Census Bureau takes the ZIP code used by the majority of areas in each census unit at the time the data are compiled.

A ZCTA is designated as an Urban Area or Urban Cluster (UA/UC) based on the 2010 Census. We defined “rural” as those ZCTA areas in which 95% or more of addresses were in census-designated non-UA or non-UC areas. Based on this classification, 45.2% of veterans in the current study resided in rural areas. For analysis purposes, we also varied this percent classification from 95 to 99% to estimate impact of changing this rural vs. urban categorization on study outcomes. While this classification method has limitations, in largely rural areas evidence suggests that it likely produces more accurate results [29]. Over the past decade, ZCTA data have been used extensively in the US in research [30–33].

### Electronic health record (EHR) and survey data

Veterans for this study were initially identified by a veterans’ registry started at Geisinger in 2006, whereby all adult outpatients were asked about their military service and deployment history during their routine office visits [2]. Using this registry, veterans were randomly selected and recruited for this study, if they had one or more warzone deployments based on military records supplied by the veteran [1]. Patients who were institutionalized, cognitively impaired, or too ill to complete the survey were excluded. Following patient consent, trained interviewers administered structured health interviews by telephone from February 2016 through February 2017. Among veterans selected, all were under 76 years of age and served in Vietnam and/or another post-Vietnam conflict (i.e., Iraq/Afghanistan, Global War on Terrorism, Persian Gulf War, or another recent conflict). We were able to complete 1730 interviews with an estimated survey cooperation rate of 55% among those eligible for the survey [1]. Altogether, 38 veterans were missing zipcode information (due to moving or having only a post-office box) and were excluded from some analyses. The average survey time for the current interview was 65 min. Using data in the patient’s EHR, and with IRB approval, we examined potential response bias between survey respondents and non-respondents in terms of gender, race, age, marital status, smoking status, and the prevalence of common medical conditions. The only significant differences found were that survey respondents tended to be younger and more often married (*p <* 0.05).

All interviewers for this study had prior survey experience and received additional training by the study team on administration of the survey protocol, which included use of emergency mental health procedures and referrals. All surveys for this study took place in Geisinger’s Survey Center in Danville, PA and all interviewers were closely monitored by both the survey center staff and the study team.

### Data collection instruments

The data-collection framework included assessment of pre-trauma, within trauma, and post-trauma risk and protective factors, as well as expected health outcomes [34–36]. The outcomes included PTSD, major depression disorder, alcohol misuse, global mental health severity, and mental health treatment. We also collected risk factor data related to the veteran’s military history, concussion exposure, combat/trauma exposure, current life stressors, demographic background, and psychosocial factors, such as current social support and level of current social capital.

PTSD was assessed using a questionnaire based on the Diagnostic and Statistical Manual of Mental Disorder, Fifth Edition, The PTSD Checklist for DSM-5 (PCL-5) [37, 38]. This PTSD scale has been used in several recent studies [1, 39–41]. The diagnosis was based on the past 12 months.

Depression was assessed using a major depressive disorder scale based on the DSM-IV diagnostic criteria [42–44], which has been used in previous trauma studies [45, 46], including in the National Women’s Study among others [47, 48]. Previous studies supported the validity of this scale in diagnosing depression [47, 48]. The diagnosis was based on the past 12 months.

Other post-deployment health outcomes included measures from the Brief Symptom Inventory-18 (BSI-18) scale [49]. These mental health symptoms were assessed for the past 30 days. The BSI-18 scale is a widely used psychological symptom scale originally developed from the Hopkins Symptom Inventory, and later extensively used in research [50–53]. In the current study, we present the results for the BSI Global Severity Index, which is considered a good overall measure of mental health severity [49]. We used the standard BSI-18 cutoff score of 65 or higher to define a high Global Severity [49].

Alcohol misuse was assessed using the AUDIT-C instrument, a widely used and validated scale often used in population health studies [54]. The AUDIT-C has been found to be a good measure of alcohol misuse [55, 56]. We used the recommend AUDIT-C score of 4 for men and 3 for women to define a case for alcohol misuse [56].

We also included questions related to the use of mental health service in the past 12 months, as described previously [48, 57]. This health services utilization measure was from the National Comorbidity Survey [58], and also used in the World Mental Health (WMH) version of the World Health Organization (WHO) Composite International Diagnostic Interview (WMH-CIDI) [59]. The study team has also used these utilization measures in past trauma studies, including studies of Hurricane Sandy in New Jersey [46] and mental health service use after the World Trade Center attacks in New York City [48, 57, 60]. In addition to this measure, we also included similar survey measures related to VA service use, both current and lifetime [2]. The service use survey questions asked: “Did you ever in your lifetime [past year] go to see any of the following professionals or self-help groups for problems with your emotions or nerves or for problems with your use of alcohol or drugs?” This survey question was followed by a list of professional service providers, including psychiatrists, medical doctors, psychologists, social workers, and ministers (the detailed study survey questions are available from the study PI [JAB] upon request).

The pre-trauma, within trauma, and post-trauma risk and protective factors included demographic factors (e.g., age, gender, race, marital status, and education), number of warzone deployments, concussion history, and combat/other trauma exposures [2, 26]. Combat exposure was based on the Combat Experience Scale (CES), a widely used measure of combat exposure that has been utilized since the Vietnam War [61]. Versions of this scale have been used over the past 30 years [62–64]. The scale measures for combat exposure were divided into cut-off points (high vs. low) described elsewhere [2, 26].

Our study also assessed the occurrence of lifetime traumatic events (e.g., forced sexual contact, domestic abuse, a serious accident, warzone exposure, major disaster exposure) using a World Trade Center Disaster lifetime trauma scale [65] developed from previous trauma research [36]. This scale has been used in numerous studies since the 9/11 attacks [2, 46, 60, 66–70]. Since we had no *priori* method to judge the severity of these events, these exposures were collapsed into three categories: less than 3 traumatic events, 3–5 events, and 6 or more events. The reported reliability and validity of this trauma scale is good [2, 36, 46, 65, 66, 71].

The current study also included measures of current life stressors, current social support, and VA service use, all based on survey questions [2]. Current life stressors included a count of eight experiences that could have happened to the respondent in the past 12 months (e.g., death of spouse or close family member, being injured, problems at work, getting married, having financial problems, etc.). Experiencing two or more of these events was classified as having high exposure to stressful life events [2]. Similar to the traumatic event scale, this stressful life events scale was developed from other trauma studies, used in previous research [2, 46, 60, 66–70], is reported to have good reliability and validity [36, 46, 65, 66, 71].

The social support scale used was a version included in the Medical Outcomes Study [72], that was also used in past trauma research [2, 36, 46, 60, 65–70]. This scale is considered a reliable and valid measure of current social support [48, 65]. Low social support was defined as the lowest quartile [66].

We also asked veterans about their neighborhood and the connections they have with their neighbors. The six items from the Social Capital Scale were used for this, which inquire if the neighborhood is a good place to live, if the respondent expects to live in the neighborhood for a long time, etc. This scale was from the General Social Survey, a widely use ongoing national survey in the United States [73]. We summed these questions and divided respondents into low social capital versus high social capital, with higher scores used to define higher social capital.

Concussion history was assessed based on reported concussions experienced during military service (e.g., ever dazed, confused, saw stars, or knocked out) [74], which is a concussion scale that has been used and validated in previous research [1, 2, 13].

### Data analyses

Statistical analyses included descriptive statistics, which depicted the study population and assessed the association between mental health status and rural residence. In multivariate logistic regression, key risk/protective factors (e.g., combat exposure, lifetime trauma exposure, number of deployments, and rural residency) were used to estimate the likelihoods (i.e., odds ratios, ORs) for PTSD, depression, alcohol misuse, Global Severity Index, and mental health service use, respectively. Potential confounding factors included age, gender, marital status, and level of education. To evaluate if current service use was affected by current mental health status, rather than access to care problems or convenience issues [75], we also included current mental status (i.e., current PTSD, depression, alcohol misuse, and global severity) in the regression model for service use. Subgroup analyses were conducted based on rural vs nonrural residence for this outcome.

Electronic health record was used to handle demographic data missing from the survey. For missing data on the BSI-18 scale, we used the method commonly utilized with missing psychometric scale data, whereby the non-missing items for the scale/subscale for the subject are included in the score; however, if the majority of items are missing for the subject, then the entire scale/subscale is defined as missing [49]. For psychiatric categories (e.g., PTSD, depression, alcohol misuse), we used symptom count data and ignored the refuse and no answer categories [48, 65]. For some analyses, it was necessary to excluded veterans that only reported Post Office boxes and/or those who moved out of state before the survey began (*n* = 38). Sample size estimate was based on a pilot study showing that 10–15% of Geisinger veterans developed mental disorders following deployments (including PTSD, depression, and alcohol misuse) and that most of their associated risk factors were at least moderately elevated [2]. Using n-Query Advisor, Version 5.0, [76], we estimated that a sample size of 1500 would be adequate for most hypothesis testing in our study (*N =* 1692), assuming a two-sided alpha = 0.05, at 80% power, and moderate effect sizes (*d =* 0.35). Given the expanding service options available for veterans [25, 77], we expected the findings for rural veterans to be better than previously reported, but this assumption was tentative. Analyses were conducted using Stata, version 15.1 software [78].

## Results

Majority of the veterans were 65 years of age or older (56.4%), male (95.4%), White (95.7%), and currently married (77.7%). In addition, 24.4% were college graduates, and 39.7% were deployed on multiple tours (Table 1). Moreover, 8.3% met the criteria for depressive disorder in the past year, 7.7% met the criteria for PTSD, 24.3% had a positive alcohol screen on the AUDIT-C scale, 49.9% were currently using VA services, and 23.5% utilized psychiatric services (either in the VA or elsewhere) in the past year (Table 2). Analyses comparing demographic, medical, and psychological variables among veterans residing in rural versus nonrural areas also revealed several significant differences (Table 1). Veterans residing in rural areas tended to be older (65 of age and above) (*OR =* 1.24, *P =* 0.028; 59.3% vs 54.0%), more often White (*OR =* 1.69, *P =* 0.034; 96.9% vs 94.8%), more often married (*OR =* 1.44, *P =* 0.002; 81.1% vs 74.9%), less often college graduates (*OR =* 0.69, *P =* 0.001; 20.7% vs 27.5%), have experienced fewer stressful events in the past year (*OR =* 0.77, *P =* 0.026; 18.9% vs 23.4%), and less often reported low social capital (*OR =* 0.59, *P <* 0.001; 15.5% vs 23.7%) (Table 1). In addition, veterans residing in rural areas had lower Global Severity Index scores (*OR =* 0.65, *P =* 0.003; 10.2% vs 14.9%) and used psychiatric services less often within the past year (*OR =* 0.65, *P <* 0.001; 19.3% vs 26.9%) (Table 2).

**Table 1:** Rural vs. non-rural residence by study predictor variables (*N =* 1692)^a^

Item	Study variables [*n*(%)]^a^	Residence^a#^		*OR*	*P*
		Non-rural (%)	Rural (%)		
^b^Age					
	18–64 [733(43.6)]	46.0	40.7	1.00	0.028
	≥65 [949(56.4)]	54.0	59.3	1.24	–
Gender					
	Male [1614(95.4)]	94.9	96.0	0.79	0.308
	Female [78(4.6)]	5.1	4.0	1.00	–
Race					
	White [1620(95.7)]	94.8	96.9	1.69	0.034
	Non-White [72(4.3)]	5.2	3.1	1.00	–
Marital status					
	Married [1315(77.7)]	74.9	81.1	1.44	0.002
	Not married [377(22.3)]	25.1	18.9	1.00	–
Education					
	College graduate [413(24.4)]	27.5	20.7	0.69	0.001
	Non-college graduate [1279(75.6)]	72.5	79.3	1.00	–
Income					
	≥$100 K [373 (22.0)]	23.1	20.7	0.87	0.234
	<$100 K [1319(78.0)]	76.9	79.3	1.00	–
Deployed tours					
	Multiple [671(39.7)]	38.7	40.9	1.10	0.364
	Single [1018(60.3)]	61.3	59.1	1.00	–
Stressful events past year					
	Yes [361(21.3)]	23.4	18.9	0.77	0.026
	No [1331(78.7)]	76.6	81.1	1.00	–
Combat exposure					
	High [403 (23.8)]	23.5	24.3	1.05	0.704
	Low [1289(76.2)]	76.5	75.7	1.00	–
Lifetime trauma					
	High [351(20.7)]	20.5	21.0	1.03	0.820
	Low [1341(79.3)]	79.5	79.0	1.00	–
Social support					
	Low [309(18.3)]	18.7	17.7	0.94	0.606
	Not low[1383(81.7)]	81.3	82.3	1.00	–
Social capital					
	Low [338(20.0)]	23.7	15.5	0.59	< 0.001
	Not low [1354(80.0)]	76.3	84.5	1.00	–

^a^Based on column percent. ^#^Proportional difference *P <* 0.001, based on 99% exact confidence intervals for nonrural vs. rural. ^b^The age had 10 missing values. Altogether, 38 veterans had missing zipcode data and excluded from this analysis

**Table 2:** Rural vs. non-rural residence by mental health status measures (*N =* 1692)

Item	Study variable (totals) [*n*(%)]	Residence^a^		*OR*	*P*
		Non-rural (%)	Rural (%)		
^b^Global severity (BSI-18)					
	High [213(12.8)]	14.9	10.2	0.65	0.003
	Not high [1457 (87.2)]	85.1	89.8	1.00	–
Depression					
	Current depressive disorder [141 (8.3)]	8.6	8.0	0.91	0.606
	No current depressive disorder [1551 (91.7)]	91.4	92.0	1.00	–
PTSD					
	Yes in past year [130 (7.7)]	8.8	6.4	0.71	0.067
	No in past year [1562 (92.3)]	91.2	93.6	1.00	–
Alcohol use					
	Positive AUDIT-C Alc. screen [411 (24.3)]	25.8	22.4	0.83	0.103
	Negative AUDIT-C Alc. screen [1281 (75.7)]	74.2	77.6	1.00	–
Head trauma					
	In-service concussion [480 (28.4)]	28.5	28.2	0.98	0.863
	No in-service concussion [1212 (71.6)]	71.5	71.8	1.00	–
VA service					
	Currently using [845 (49.9)]	49.4	50.6	1.05	0.629
	Not currently using [847 (50.1)]	50.6	49.4	1.00	–
Psych service (any)					
	Used psych services in past year [397 (23.5)]	26.9	19.3	0.65	< 0.001
	No psych services in past year [1295 (76.5)]	73.1	80.7	1.00	–

^a^Based on column percent. ^b^For global severity *n =* 1670 due to missing data on this measure. Altogether, 38 veterans had missing zipcode data and excluded from this analysis

In multivariable analyses (Table 3), significant predictors of PTSD were female sex (*OR =* 2.86), high combat exposure (*OR =* 2.70), history of concussion (*OR =* 3.42), high stressful events within the past year (*OR =* 4.15), high lifetime trauma exposure (*OR =* 2.45), low social support (*OR =* 1.77), and low social capital (*OR =* 2.11). Additionally, serving on multiple tours was found to be a protective factor for PTSD (*OR =* 0.55). Regarding significant variables predicting major depressive disorder, age was found to be protective (*OR =* 0.97), while having high combat exposure (*OR =* 1.90), history of concussion (*OR =* 2.47), high stressful life events within the past year (*OR =* 2.81), and low social support (*OR =* 3.30) were significant risk factors (Table 3). Significant risk factors predicative of higher AUDIT-C scores were younger age (*OR =* 0.96) and female sex (*OR =* 0.46), both of which were protective for alcohol misuse. In terms of Global Severity, rural residence was found to be a significant protective factor (*OR =* 0.71). Conversely, significant risk factors for high Global Severity were high combat exposure (*OR =* 1.93), history of concussion (*OR =* 2.55), high stressful life events within the past year (*OR =* 3.55), high lifetime trauma exposure (*OR =* 2.08), low social support (*OR =* 2.90), and low social capital (*OR =* 1.61). Significant factors associated with receiving psychiatric treatment in the past year were rural residences (*OR =* 0.69), and age (*OR =* 0.98), both protective. Conversely, female sex (*OR =* 2.79), high combat exposure (*OR =* 1.98), history of concussion (*OR =* 2.68), high stressful life events in the past year (*OR =* 2.51), high lifetime trauma exposure (*OR =* 2.03), low social support (*OR =* 1.52), and low social capital (*OR =* 1.78) were positively associated with having received psychiatric treatment in the past year. After adjusting for potential confounders, rural residence was negatively associated with both high global severity and service use in the past year (Table 3). Rural residence was also negatively associated with current health service use after adjustment for mental health status (*OR =* 0.70, *P =* 0.013; Table 4).

**Table 3:** Multivariable logistic regressions predicting current PTSD, depression, AUDIT-C, global severity index, and psychiatric service utilization among veterans (*N =* 1666 ~ 1692) [OR, 95% CI]*

Variables	PTSD	Major depression	AUDIT-C positive	High global severity (BSI-18)	Psych treatment past year
Rural residence	0.91, 0.60–1.39	1.11, 0.76–1.63	0.90, 0.72–1.14	0.71^†^, 0.51–0.99	0.69^‡^, 0.53–0.90
Age (in years)	0.98, 0.97–1.00	0.97^§^, 0.96–0.98	0.96^§^, 0.95–0.97	1.00, 0.98–1.01	0.98^§^, 0.97–0.99
Female sex	2.86^†^, 1.26–6.45	1.99, 0.96–4.10	0.46^‡^, 0.26–0.82	1.73, 0.83–3.58	2.79^§^, 1.62–4.80
White race	1.89, 0.64–5.60	0.76, 0.34–1.70	0.76, 0.45–1.29	2.14, 0.83–5.55	1.15, 0.62–2.12
Income over $100 K	0.99, 0.60–1.64	0.83, 0.52–1.34	1.11, 0.85–1.46	0.69, 0.45–1.06	0.85, 0.62–1.16
Multiple tours	0.55^‡^, 0.35–0.86	0.91, 0.62–1.35	0.86, 0.67–1.09	0.80, 0.57–1.13	1.03, 0.79–1.34
Combat high	2.70^§^, 1.73–4.20	1.90^‡^, 1.24–2.91	1.00, 0.75–1.34	1.93^§^, 1.34–2.77	1.98^§^, 1.48–2.66
Concussion history	3.42^§^, 2.19–5.34	2.47^§^, 1.64–3.72	1.01, 0.77–1.34	2.55^§^, 1.80–3.62	2.68^§^, 2.03–3.55
High stress past year	4.15^§^, 2.71–6.37	2.81^§^, 1.89–4.19	1.02, 0.76–1.37	3.55^§^, 2.51–5.01	2.51^§^, 1.89–3.34
High life trauma	2.45^§^, 1.60–3.74	1.31, 0.86–1.99	1.20, 0.91–1.60	2.08^§^, 1.46–2.96	2.03^§^, 1.52–2.71
Low social support	1.77^†^, 1.13–2.79	3.30^§^, 2.21–4.92	1.28, 0.96–1.72	2.90^§^, 2.04–4.14	1.52^‡^, 1.11–2.07
Low social capital	2.11^‡^, 1.36–3.27	1.30, 0.85–1.99	0.96, 0.72–1.29	1.61^†^, 1.12–2.33	1.78^§^, 1.32–2.40

† *P <* 0.05; ‡ *P <* 0.01; § *P <* 0.001; * Model *N =* 1666 ~ 1692 due to missing data

**Table 4:** Multivariable logistic regression predicting any psychiatric service utilization controlling for current PTSD, depression, AUDIT-C positive status, and high global severity among veterans (*N* = 1666)^a^

Variables	OR	95% CI	*P*
Age (in years)	0.98	0.97–0.99	0.003
Female Sex	2.89	1.59–5.26	0.001
White Race	1.10	0.56–2.15	0.793
Income Over $100 k	0.90	0.64–1.26	0.532
Multiple Tours	1.15	0.86–1.54	0.341
Combat High	1.68	1.21–2.33	0.002
Concussion History	2.19	1.61–2.99	< 0.001
High Stress, Past Year	1.75	1.26–2.43	0.001
High Lifetime Trauma	1.82	1.32–2.52	< 0.001
Low Social Support	1.03	0.72–1.48	0.886
Low Social Capital	1.54	1.11–2.14	0.010
High Global Severity (BSI-18)	2.50	1.59–3.93	< 0.001
AUDIT-C Positive	0.90	0.65–1.25	0.517
Current PTSD	3.17	1.79–5.61	< 0.001
Current Major Depression	6.73	3.90–11.62	< 0.001
Rural Residence	0.70	0.52–0.93	0.013

^a^Model *N =* 1666 due to missing data

In comparison with those in rural areas, female veterans in nonrural areas had an increased risk for PTSD (*OR =* 3.76, *p =* 0.01), increased risk for depression (*OR =* 2.74, *p =* 0.028), increased likelihood of service use (*OR =* 3.55, *p* < 0.001), and decreased risk for alcohol problems (*OR =* 0.33, *p =* 0.004). Detailed results are available from the study PI [JAB] upon request.

## Discussion

Rural veterans often travel farther distances than urban veterans to access health care and have more limited access to quality care [6, 9, 10]. A previous study reported that rural VA patients are less likely than urban patients to have received a major mental illness diagnosis (e.g., bipolar, schizophrenia), but more likely to have had depression, PTSD, or have received an anxiety disorder diagnosis [79]. In contrast, others found no differences between rural and urban residence in frequent mental distress utilizing the Behavioral Risk Factor Surveillance System (BRFSS) survey [80]. More recently, it was reported that approximately 20% of service users live in a rural setting, and those who live in rural areas tend to live longer, but there was no difference after age 75 [81]. Specifically, it was found that rural veterans were better off than urban veterans regarding surviving to age 65, but this advantage diminished by age 75 [81].

In the current study, rural residence was not negatively associated with mental health status. However, one exception found was the negative association between rural residence and use of psychiatric services in the past year (Table 3 and Table 4), suggesting rural veterans are less likely to use these services. Another was that rural residence was negatively associated with having high Global Severity score. Noteworthy is that a recent study reported that both rural and urban veterans are increasingly making use of mental health services, and that rural-urban gaps in psychotherapy use are shrinking [82]. Nevertheless, when we examined these outcomes by rural vs. nonrural residence separately, female veterans in nonrural areas were more likely to have current PTSD, depression, and mental health service use, but less likely to have current alcohol misuse compared to rural veterans.

Our study has several limitations. First, this is a cross-sectional survey. As such, it is impossible to determine the potential cause-effect relationship [83]. For example, having PTSD may have resulted in lower current social support not the reverse. Second, study subjects were mostly White patients in central and northeastern Pennsylvania. Thus, it may not be possible to generalize these findings to other geographic areas and study populations. As noted elsewhere, however, there are few stable national samples of veterans available, since this population is dynamic, due to different deployments, ongoing conflicts, and the aging of the veteran population [19, 22, 84]. In addition, many veterans do not use the VA system for routine health care [2], which complicates identifying representative samples of veterans for clinical research. Another shortcoming is that US Census zip-code tabulation area (ZCTA) data has limitations, in that it may not accurately represent the study population under investigation [85]. To assess this, we varied the percent ZCTA classification cut points from 95 to 99% and reassessed these results. While this changed the percent rural residence classification, our results were similar, nevertheless. Finally, responders tended to be younger and more often married than non-responders, and may have biased the results.

In summary, we failed to show poorer mental health outcomes post-deployment in rural veterans. Veterans residing in rural areas tended to be older, more often White, married, experienced fewer stressful events in the past year, and less often had low social capital compared to nonrural areas. In addition, veterans in rural areas had lower Global Severity, used psychiatric services less often within the past year. In multivariable analyses, significant predictors of PTSD, depression, Global Severity, and mental health service use were generally consistent with past studies [2, 36, 48]. We speculate that the lack of difference between rural and nonrural veterans may reflect the improvements in community-based services for veterans in rural areas due to the implementation of the VA Choice program [25, 86].

## Conclusions

With the exception of alcohol misuse, adverse post-deployment outcomes were typically associated with pre-trauma, within trauma, and post-trauma factors related to stressful life events, past trauma exposures, current social support, as well as other factors [1] and this was less so with rural residency. Consistent with a previous study [87], female sex was associated with PTSD. Our results suggest that rural residence is not a post-deployment risk factor for poor mental health outcomes, but rather protective. This finding needs to be verified in future studies.

## Data Availability

The dataset used in this study analysis is not currently available.

## Abbreviations

AUDIT: Alcohol use disorders identification test
BSI: Brief symptom inventory
DoD: Department of Defense
DSM-5: Diagnostic and statistical manual of mental disorders, Fifth edition
EHR: Electronic health record
PCL-5: PTSD checklist, version 5
PTSD: Post-traumatic stress disorder
VA: Veterans affairs
ZCTA: Zip code tabulation areas
